# Angiotensin II Facilitates Breast Cancer Cell Migration and Metastasis

**DOI:** 10.1371/journal.pone.0035667

**Published:** 2012-04-20

**Authors:** Sylvie Rodrigues-Ferreira, Mohamed Abdelkarim, Patricia Dillenburg-Pilla, Anny-Claude Luissint, Anne di-Tommaso, Frédérique Deshayes, Carmen Lucia S. Pontes, Angie Molina, Nicolas Cagnard, Franck Letourneur, Marina Morel, Rosana I. Reis, Dulce E. Casarini, Benoit Terris, Pierre-Olivier Couraud, Claudio M. Costa-Neto, Mélanie Di Benedetto, Clara Nahmias

**Affiliations:** 1 Inserm, Institut Cochin, Paris, France; 2 CNRS, Paris, France; 3 Université Paris Descartes, Paris, France; 4 Université Paris 13, Bobigny, France; 5 CNRS, UMRS 940, IGM, Paris, France; 6 Department of Biochemistry and Immunology, Faculty of Medicine at Ribeirão Preto, University of São Paulo, Ribeirão Preto, Brazil; 7 Department of Physiological Sciences, Federal University of São Carlos, São Carlos, São Paulo, Brazil; 8 Department of Medicine, Nephrology Division, Federal University of São Paulo, São Paulo, São Paulo, Brazil; Emory University, United States of America

## Abstract

Breast cancer metastasis is a leading cause of death by malignancy in women worldwide. Efforts are being made to further characterize the rate-limiting steps of cancer metastasis, i.e. extravasation of circulating tumor cells and colonization of secondary organs. In this study, we investigated whether angiotensin II, a major vasoactive peptide both produced locally and released in the bloodstream, may trigger activating signals that contribute to cancer cell extravasation and metastasis. We used an experimental *in vivo* model of cancer metastasis in which bioluminescent breast tumor cells (D3H2LN) were injected intra-cardiacally into nude mice in order to recapitulate the late and essential steps of metastatic dissemination. Real-time intravital imaging studies revealed that angiotensin II accelerates the formation of metastatic foci at secondary sites. Pre-treatment of cancer cells with the peptide increases the number of mice with metastases, as well as the number and size of metastases per mouse. In vitro, angiotensin II contributes to each sequential step of cancer metastasis by promoting cancer cell adhesion to endothelial cells, trans-endothelial migration and tumor cell migration across extracellular matrix. At the molecular level, a total of 102 genes differentially expressed following angiotensin II pre-treatment were identified by comparative DNA microarray. Angiotensin II regulates two groups of connected genes related to its precursor angiotensinogen. Among those, up-regulated MMP2/MMP9 and ICAM1 stand at the crossroad of a network of genes involved in cell adhesion, migration and invasion. Our data suggest that targeting angiotensin II production or action may represent a valuable therapeutic option to prevent metastatic progression of invasive breast tumors.

## Introduction

The occurrence of distant metastasis is a critical event that limits the survival of patients with breast cancer. While targeted molecular therapies have considerably improved the management of primary breast tumors, these remain poorly effective for the treatment of distant metastases. The identification of molecular agents that may contribute to breast cancer cell dissemination is therefore essential for future development of new anti-metastatic therapeutic strategies.

Metastasis is an inefficient process. Among the large number of cancer cells that detach from the primary tumor and invade adjacent tissues to reach the bloodstream, most remain quiescent or die in the circulation [1]–[3]. Only few circulating tumor cells are able to cross the blood barrier and colonize distant organs to form micrometastases [3]–[5]. There is increasing evidence that, in addition to intrinsic metastasis gene signatures that predict the ability of tumor cells to colonize distant tissues [6], close interactions between circulating tumor cells and the host microenvironment are critical to the establishment of cancer cells at secondary sites [7]–[9]. Diffusible molecules such as cytokines or chemokines (CXCL12, CCL2) play a seminal role in breast cancer metastasis [10], [11]. We reasoned that other small molecules such as vasoactive peptides, either produced locally or released in the blood flow, may trigger activating signals contributing in an autocrine or paracrine manner to cancer cell extravasation, colonization and metastasis.

Angiotensin II (AngII) is the biologically active peptide of the renin-angiotensin system (RAS) involved in blood pressure control, tissue remodeling and angiogenesis as well as in vascular and inflammatory pathologies. Of interest, major functions attributed to AngII (inflammation, angiogenesis and migration) are also related to cancer progression [12], [13]. Most components of the RAS including angiotensinogen, angiotensin converting enzyme (ACE) and angiotensin receptors are expressed locally in a wide variety of tumors, including in breast tumors [13]–[15]. Local production of AngII in gastric cancer has been shown to facilitate tumor progression and lymph node metastasis [16], [17]. Furthermore, blockers of the RAS (either ACE inhibitors or angiotensin receptor blockers ARBs) were shown to efficiently reduce tumor growth, angiogenesis and metastasis in mouse experimental models *in vivo* [12], [13], [18], [19]. However, anti-metastatic properties of RAS inhibitors were mainly associated with effects on the host microenvironment, including infiltration of tumor-associated macrophages or tumor-related angiogenesis [20], [21], and to date there has been no report on potential metastatic effects of AngII through direct cancer cell activation.

In this study, we aimed to investigate whether AngII may act directly on tumor cells to modify their metastatic properties. We demonstrate that pre-treatment of breast cancer cells by AngII triggers rapid development of metastatic foci at secondary sites in an experimental mouse model *in vivo* and potentiates cancer cell motility and transendothelial migration.

## Results

### Angiotensin II accelerates the development of metastases *in vivo*

An experimental mouse model of cancer metastasis was developed to investigate the effects of AngII on the metastatic potential of breast cancer cells *in vivo*. Highly metastatic human breast cancer cells D3H2LN (an *in vivo*-selected subclone of MDA-MB-231 cells expressing luciferase [22]) were exposed to AngII (100 nM) for 24 hrs (or vehicle for control group) and injected intra-cardiacally into the bloodstream of nude mice in order to recapitulate the late and essential steps of cancer metastasis, i.e. extravasation and colonization [22], [23]. Such strategy allowed us to evaluate the effects of AngII on cancer cells while avoiding any direct effect of the peptide on the host microenvironment.

**Figure 1:**
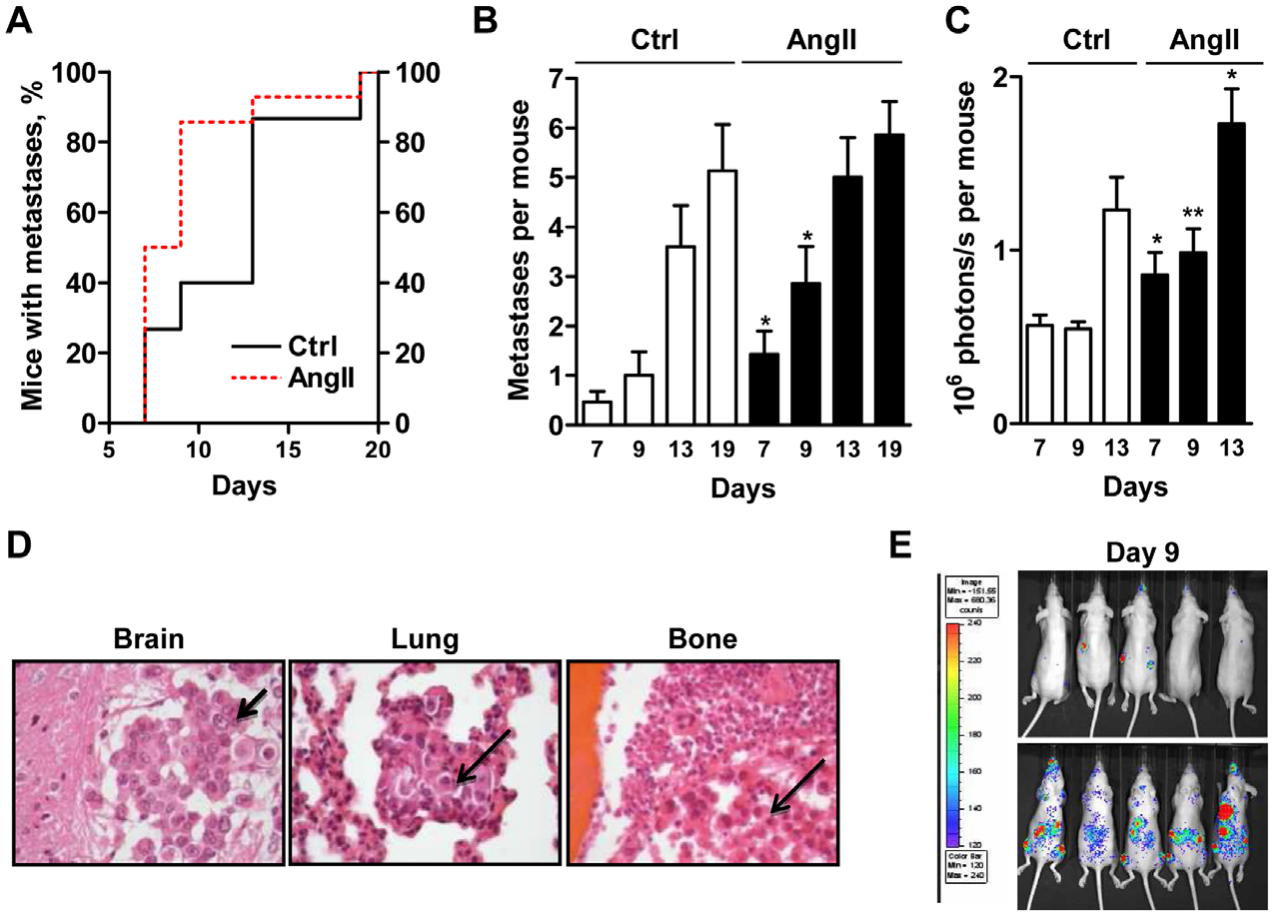
AngII increases the time-course, incidence and number of metastases in an experimental model *in vivo*. (**A**). Percentage of mice showing at least one detectable metastasis over time after intracardiac injection of D3H2LN cells treated with AngII (red dotted line, n = 14) or vehicle (black line, n = 15). (**B**). Number of metastases per mouse at indicated days. Results are mean +/− SEM of 15 control (white bar) and 14 AngII-treated (black bar) groups. (**C**). Number of photons/s per mouse at indicated days. Results are expressed as in B. (**D**). Histological analysis of metastases developing at the brain (left panel), the lung (middle panel) and the bone (right panel), obtained from 3 µm sections of formalin-fixed, paraffin-embedded tissue blocks stained with hematoxylin/eosin. Arrows indicate tumor cells. Magnification, 200x. (**E**). Representative pictures of 5 mice taken at day 9 after injection of control cells (upper panel) or AngII-treated cells (lower panel). * p<0.05, ** p<0.01.

The establishment of tumor micrometastases in various organs was evaluated every two days by intravital bioluminescent imaging on anesthetized animals. Fourteen mice injected with AngII-treated cells were compared to 15 control mice, in two independent experiments. As shown in Fig. 1A, mice from both groups showed detectable micrometastases as early as day 7 post-injection and all of them harbored metastases at day 19, illustrating high aggressiveness of the D3H2LN cell line. However, tumor cells exposed to AngII acquired a more aggressive behavior, showing at least one metastatic site in 50% (7/14) of the animals at day 7 as compared to 26,7% (4/15) of control mice. At day 9 of the experiment, 86% (12/14) of the mice that received AngII-treated cells presented at least one detectable metastatic nodule, compared to 40% (6/15) for control mice (Fig. 1A). Notably, AngII pre-treatment not only increased the percentage of mice with metastasis, but also increased the number of detectable metastatic foci per mouse (Fig. 1B) as well as the total number of tumor cells disseminated in the whole body, as assessed by quantification of bioluminescence (Fig. 1C). *Ex-vivo* analysis of bioluminescence in isolated organs (not shown) and subsequent histological analysis (Fig. 1D) on the last day of the experiment confirmed the presence of tumor cells in the brain, lung and bone samples that had been identified as luciferase-positive in the whole animal.

The most significant differences between AngII-pretreated and control groups were observed shortly after cell injection, as illustrated by pictures of 5 representative mice taken at (Fig. 1E). Indeed, breast cancer cells treated with AngII developed three times more metastatic foci per mouse at day 9 compared to control cells (Fig. 1B, Fig. S1A). In agreement, the number of disseminated cancer cells was significantly increased in the AngII-treated group as compared to control (median 1.155 and 0.525×10^6^ of photons/s respectively, at day 9 post-injection) (Fig. 1C, Fig. S1B). Our results thus indicate that invasive D3H2LN breast cancer cells exposed to AngII show increased metastatic potential *in vivo* and are more prone to rapidly establish at distant organs.

### Angiotensin II increases breast cancer cell adhesion and migration

**Figure 2:**
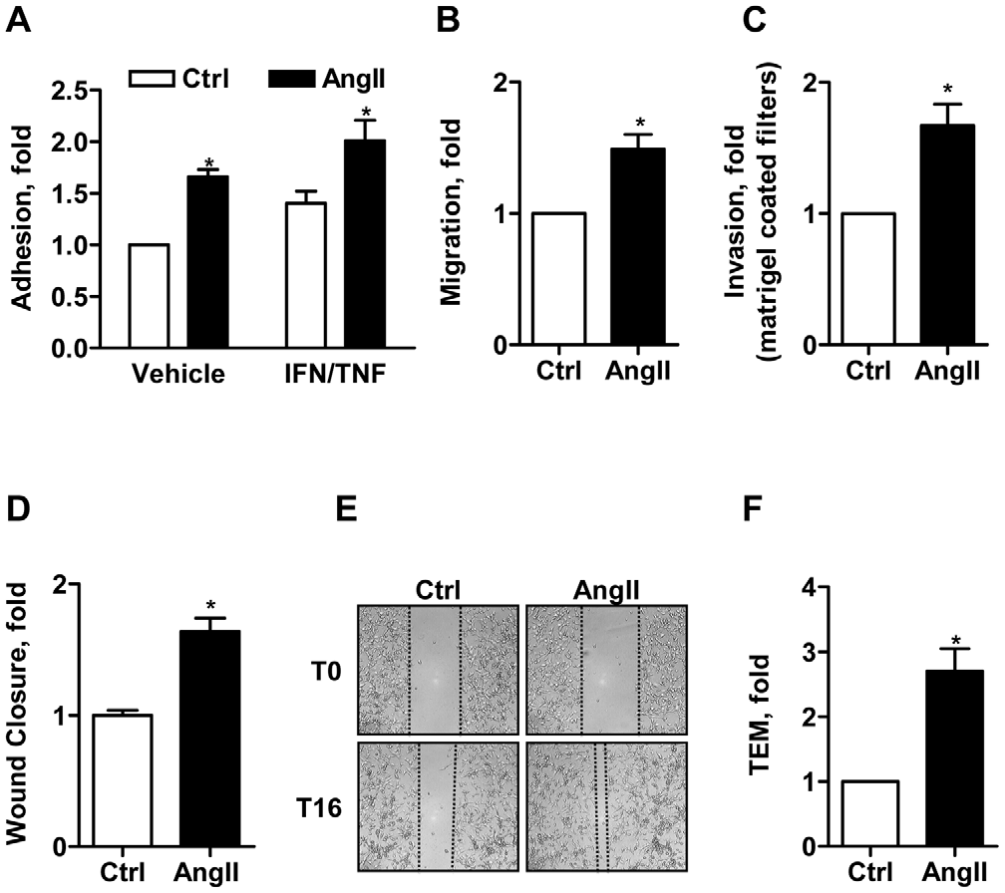
AngII increases breast cancer cell adhesion and migration. (**A**). MDA-MB-231 breast cancer cell adhesion to HCMEC/D3 endothelial cells monolayer following exposure of cancer cells to AngII (100 nM) for 24 hrs. Results are means +/− SEM of 7 independent experiments performed in quadruplicate, and expressed as fold increase of untreated cells (control, Ctrl). *p<0.05. (**B, C**). Boyden chamber assays of tumor cell migration across 8 µm-pore filters either non coated (B) or coated with matrigel to mimic cell invasion (C). Results are means +/− SEM of 3 separate experiments performed in triplicate, and expressed as fold increase of control. *p<0.05. (**D, E**). Wound healing assay. Results are from 2 independent experiments performed in quintuplicate, and expressed as fold increase of wound closure at time 16 hrs (T16) compared to control (vehicle-treated cells). *p<0.05. (E). Representative pictures of wounds from control and AngII-treated cells (100 nM, 24 hrs) at T0 and T16. Magnification, 100x. (**F**). Trans-endothelial migration. Results are mean +/− SEM of 3 independent experiments performed in triplicate, and expressed as fold increase of control. *p<0.05.

Metastatic dissemination of circulating cancer cells involves several sequential steps, among which tumor cell adhesion to the vascular endothelium, migration across the endothelial barrier and subsequent invasion across the extracellular matrix to reach a secondary site. In order to evaluate the consequences of AngII activation on cancer cell adhesion and migration, the properties of MDA-MB-231 and D3H2LN breast cancer cells were analyzed *in vitro* following pre-treatment with AngII. As shown in Fig. 2A, AngII stimulation for 24 hrs significantly increased (1.7 fold) the adhesion of cancer cells to a monolayer of human endothelial cells. Cancer cell adhesion following AngII stimulation was also increased (2 fold) when endothelial cells were pre-activated for 24 hrs with pro-inflammatory cytokines (IFNγ and TNFα). To note, short-term exposure (30 min or 6 hrs) of breast cancer cells to AngII was not sufficient to promote increased adhesion to the endothelial monolayer (data not shown), suggesting that AngII-increased cancer cell adhesion may involve transcriptional regulation of target genes rather than activation of intracellular trafficking or signaling pathways – that generally occur within minutes.

We next evaluated the effects of AngII on breast cancer cell migration. As shown in Fig. 2B, pre-treatment of breast cancer cells with AngII for 24 hrs significantly increased (1.5 fold) their ability to migrate in Boyden chamber assays using FCS as chemoattractant. Similar results were obtained in invasion assays using filters coated with matrigel® that mimics the extracellular matrix (Fig. 2C). The pro-migratory effects of AngII on breast cancer cells were further confirmed in wound healing assays (Fig. 2D, E) showing significant increase (1.64 fold) in cell migration and wound closure at 16 hrs following pre-treatment with AngII. To note, AngII-pre-treatment had no significant effect on cell proliferation (Fig. S2), ruling out the possibility that increased cell number may account for increased wound closure. Finally, exposure of breast cancer cells to AngII induced a 2.7 fold-increase in trans-endothelial migration, i.e. the ability to migrate through a monolayer of human endothelial cells (Fig. 2F), which is a hallmark of cancer cell extravasation *in vitro*.

Thus, AngII contributes to each step of breast cancer cell extravasation including tumor cell adhesion to endothelial cells, motility, invasion and trans-endothelial migration.

### Angiotensin II regulates a panel of connected target genes

To get further insight into the mechanisms by which AngII increases breast cancer cell migration and metastasis, we searched for downstream molecular targets that may be regulated following exposure of MDA-MB-231 cells to AngII for 24 hrs. Comparative DNA microarray (Affymetrix U133A) studies revealed a panel of 123 differentially expressed genes (more than 1.4-fold, p<0.05). Among those, 102 genes (63 up-regulated and 39 down-regulated) were associated with known functions (Tables S1 and S2) including cell proliferation and apoptosis (32%), cell adhesion and migration (27%) and inflammation (18%) (Table S3). Accordingly, these genes were found to contribute to intracellular protein kinase pathways (21%) or small GTPase signaling (17%) (Table S4). Of interest, a large number of differentially regulated genes (25%) were also related to cell metabolism, a finding that opens new areas of investigation regarding the effects of AngII in cancer cells.

**Figure 3:**
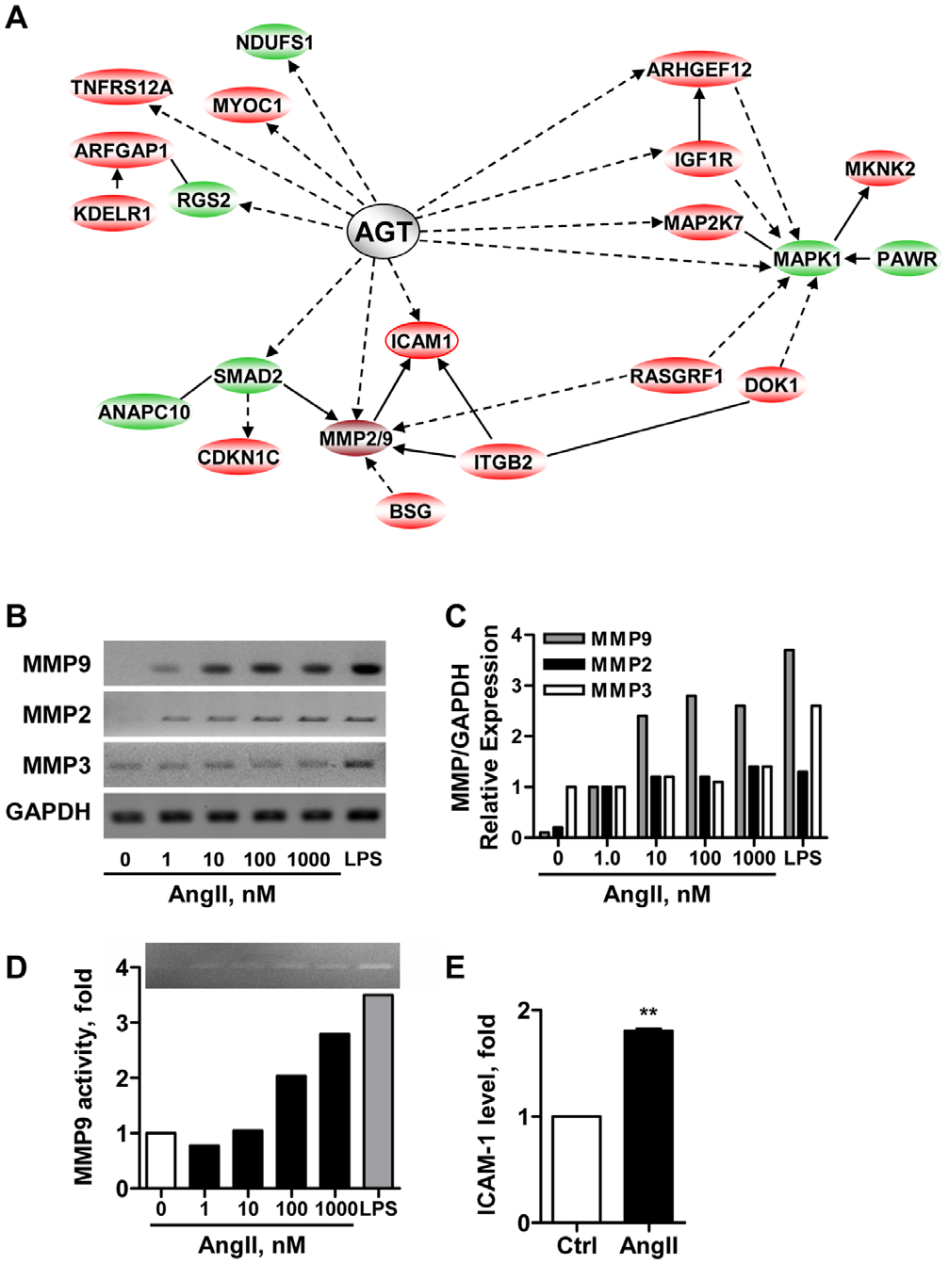
AngII transcriptionally regulates a panel of connected genes. (**A**). Gene networks differentially regulated by AngII. Up- and down-regulated genes related to angiotensinogen (AGT) are indicated in red and green, respectively. Filled lines indicate direct interactions, filled and dashed arrows indicate direct and indirect regulations, respectively. Note two groups of connected genes centered around MAPK1 and MMP2/9, respectively. (**B**). RT-PCR analysis of MMP9, MMP2 and MMP3 mRNA expression in MDA-MB-231 cells treated for 24 hrs with increasing doses of AngII as indicated, or LPS (Lipopolysaccharide, 100 ng/ml) as a positive control. GAPDH amplification was used as internal control. Shown is one out of 3 to 5 independent experiments performed in duplicate. (**C**). Quantification (Image J software) of PCR amplification of MMP9, MMP2 and MMP3 relative to GAPDH and normalized to expression levels in cells treated with 1 nM AngII. (**D**). Gelatin-based zymography analysis of MMP9 activity in conditioned medium of cells treated as in B. Shown is one representative out of 3 independent experiments (Upper panel). Quantification (ImageJ software) of results normalized to the quantity of proteins in cell lysate and expressed relative to control (lower panel). (**E**). FACS analysis of ICAM-1 expression at the plasma membrane of MDA-MB-231 cells treated with AngII (100 nM) or vehicle for 24 hrs. Results are means +/− SEM of 3 independent experiments and expressed as fold-increase of the control. **p<0.01.

Except for one up-regulated gene (encoding anti-apoptotic molecule ATAD3A), differential regulation by AngII at 24 hrs did not exceed a factor of 3 (Table S1), suggesting that AngII may induce fine-tuned modulation of a wide number of genes involved in various signaling pathways, rather than strong activation or inhibition of a restricted set of specific genes. Ingenuity Pathway Analysis (IPA) software revealed a network of genes centered around angiotensinogen (AGT), the precursor of AngII (Fig. 3A). Remarkably, two main groups of regulated genes could be distinguished, one being related to MAP kinase (MAPK1) a major effector of cell proliferation and inflammation (comprising MAPK1, MAP2K7, MKNK2, PAWR, ARHGEF12, IGF1R, RASGRF1 and DOK1), the other one being connected to matrix metalloproteases MMP2 and MMP9 (also comprising ICAM1, ITGB2, BSG, CDKN1, ANAPC10, SMAD2, RASGRF1 and DOK1), well-known mediators of cell invasion and matrix remodeling (Fig. 3A). Notably, RASGFR1 and DOK1 belong to both groups of connected genes.

To note, microarray studies indeed revealed an increase in MMP2 and MMP9 expression levels in response to AngII stimulation, although results did not reach significance due to heterogeneity of probesets hybridization. The pivotal position of these genes within the network of AngII-regulated targets prompted us to further investigate their differential expression by RT-PCR. As shown in Fig. 3B and 3C, AngII dose-dependently increases the mRNA levels of MMP2 (2-fold) and MMP9 (3-fold) but not MMP3 nor MMP1 (not shown). Lipopolysaccharide (LPS), as well-known potent inducer of MMPs expression and activity, was used as a positive control for AngII efficiency. Dose-dependent activation of MMP9 enzymatic (gelatinase) activity, reaching a 2-fold increase at 100 nM AngII, was further confirmed by zymography analysis (Fig. 3D). Of interest, Intercellular Adhesion Molecule (ICAM-1), a major player in cell-cell adhesion and trans-endothelial migration, also stands at the crossroad between AGT, MMPs and integrins (Fig. 3A). In agreement with gene array studies showing up-regulation of ICAM-1 mRNA (1.48 fold) by AngII (Table S1), FACS analyses (Fig. 3E) further confirmed up-regulation (1.8-fold) of ICAM-1 protein levels at the plasma membrane of MDA-MB-231 cells following 24 hrs treatment with AngII.

## Discussion

Pro-metastatic effects of AngII in various experimental models *in vivo* have been attributed to its actions on the host microenvironment [12], [13], [20], [21]. We show here for the first time that direct exposure of breast cancer cells to AngII contributes to increased tumor-endothelial cell adhesion, trans-endothelial migration and motility, and accelerates metastatic progression in an experimental mouse model *in vivo*. AngII is a potent vasoactive peptide that can be both released in the bloodstream and generated locally by endothelial, stromal and/or cancer cells. We propose that autocrine or paracrine effects of AngII, either present in the circulation or in the microenvironment of secondary tissue, may trigger an activating signal facilitating the dissemination and establishment of micrometastases in target organs.

Cancer cell extravasation and metastatic colonization are rate-limiting steps that involve reciprocal interactions between tumor cells and the host stroma [24]. Extravasation requires cancer-endothelial cell adhesion and subsequent trans-endothelial migration. Colonization in turn necessitates remodeling of the extracellular matrix to invade and adapt to the new microenvironment [25], as well as activation of pro-survival pathways that allow maintenance of cancer cells and their growth as micrometastases [24], [26]. Data presented here provide evidence that AngII transcriptionally modulates a wide range of coordinated genes that contribute to cell adhesion/migration and proliferation/survival through connection to matrix metalloprotease and MAP kinase pathways, respectively. These observations are in support of the functional studies reported here and suggest that AngII may contribute to both extravasation and colonization of metastatic breast cancer cells.

At the molecular level, previous studies have extensively documented AngII-mediated regulation of MAP kinase pathways in various cell types, in relation with mitogenic and anti-apoptotic effects of the peptide [27]. We show here that AngII up-regulates MMP2 and MMP9 gene expression and enzymatic activity in breast cancer cells, in agreement with studies conducted in the gastric cancer cell line MNK-28 [28]. Notably, we also report here that AngII up-regulates the expression of Intercellular Adhesion Molecule ICAM-1 at the mRNA and protein level. ICAM-1 is well-known to trigger leukocyte adhesion to the endothelium and subsequent diapedesis, and its expression in endothelial cells has been shown to be increased by AngII in inflammatory situations [29]. Our results show for the first time that ICAM-1 is up-regulated in breast tumor cells in response to AngII treatment. Relevance of this finding to human disease is supported by a recent report showing that increased levels of ICAM-1 in breast tumors are associated with a more aggressive phenotype [30], and by studies highlighting the importance of vascular cell adhesion molecules in the establishment of breast cancer cells at the secondary site [31]. Other genes encoding adhesion molecules (ITGB2) or metabolic pathways (FUT4) were also significantly up-regulated by AngII (1.5 and 1.7 fold, respectively) (Table S1). Of interest, FUT4 encodes fucosyltransferase which is involved in the synthesis of sialyl-Lewis X, a well-known ligand of selectins adhesion molecules, suggesting an indirect effect of AngII on the selectin–selectin ligand axis.

We propose here a model in which direct stimulation of circulating cancer cells by locally-produced AngII may regulate a set of genes that ultimately influence the host microenvironment to facilitate cancer cell extravasation, adaptation to the soil and subsequent metastatic colonization. This model supports the notion that targeting AngII production or action using ACE inhibitors or ARBs, respectively, may represent an interesting therapeutic option to prevent metastatic progression of invasive breast tumors. In patients however, the question of whether RAS blockers may have beneficial effects in cancer remains contradictory [12], , a finding that might reflect tumor heterogeneity in terms of RAS expression and local levels of AngII production. Future prospective studies analyzing expression of RAS components and AngII production in breast cancer may lead to the identification of a subpopulation of tumors that respond to ACE inhibitors and/or ARBs. Such agents being largely used in the clinics as antihypertensive agents with mild side effects may constitute a major breakthrough for personalized therapy of metastatic breast tumors.

## Materials and Methods

### Cell lines

MDA-MB-231-Luc-D3H2LN luciferase-positive breast cancer cells (referred here as D3H2LN) were obtained from Caliper Life Science (Xenogen, MA, USA) and grown as described previously [23]. These cells were derived from a spontaneous lymph node metastasis of the MDA-MB-231 adenocarcinoma cell line expressing luciferase, as described [22]. Metastatic MDA-MB-231 breast tumor cells were obtained and grown as described previously [36]. Human vascular endothelial HCMEC/D3 cells were immortalized from brain microcapillaries and grown as described [37].

### Animal studies

Intracardiac experimental mouse model of metastasis *in vivo* was conducted as described [22], [23]. Briefly, female nude mice of 8–0 weeks (Janvier, France) were anesthetized by intraperitoneal injection of 120 mg/kg ketamine and 6 mg/kg xylazine. D3H2LN cells expressing luciferase were pre-treated with 100 nM AngII (Sigma, France) or vehicle in serum-free medium for 24 hrs prior to injection (100.000 cells in 100 µl sterile PBS) into the left ventricle of the heart by non surgical means. Anesthetized mice were placed in the IVIS^TM^ Imaging System (Xenogen, Caliper Life Science, MA, USA) and imaged from both dorsal and ventral views five minutes after intraperitoneal injection of D-luciferin (Caliper Life Science). A successful intracardiac injection was indicated on day 0 by systemic bioluminescence distributed throughout the animal. Only mice with evidence of successful injection were included in the experiment. Assessment of subsequent metastasis was monitored by imaging using the IVIS^TM^ Imaging System (Caliper Life Science), every 3–4 days for up to 24 days on mice anesthetized by exposure to 1–3% isoflurane. Experiments were carried out with the approval of the Département d'Expérimentation Animale, Institut d'Hématologie, Hôpital St-Louis ethical committee, and were performed twice on 7 to 8 mice per group.

For ex-vivo analysis, organs highlighted by bioluminescence in whole mice were removed surgically after sacrifice of the animals and rapidly incubated with D-luciferin before imaging using the IVIS system. For histological analyses, sections (3 µm) of metastatic organs were cut from formalin-fixed, paraffin-embedded tissue blocks, counterstained with hematoxylin-eosin and examined under an inverted microscope.

### Tumor cell adhesion to endothelial cells and trans-endothelial migration

For endothelial cell adhesion assay, tumor cells were pre-treated with AngII (100 nM) or vehicle in serum-free medium for 24 hrs prior to labeling using green fluorescent cell tracker CMFDA (Molecular Probes) as recommended by the manufacturer. Fluorescent tumor cells (100.000/well of 96-well plates) were added for 30 min at 37°C to a monolayer of human endothelial cells (HCMEC/D3) either left untreated or pre-treated for 24 hrs with pro-inflammatory cytokines IFNγ (200 U/ml) and TNFα (100 U/ml). After extensive washing, adherent cells were lysed in water and tumor cells were quantified in a fluorescent microplate reader at wavelength 485/530 nm. Experiments were performed in quadruplicate.

For trans-endothelial migration assay, endothelial HCMEC/D3 cells (20.000/well) were plated on collagen type I-coated Transwell filters (8 µm pore filter) and grown to confluence. Serum starved MDA-MB-231 cells (100.000/well) were pre-treated for 24 hrs with AngII (100 nM) or vehicle prior to labeling with CMFDA cell tracker as described before. Fluorescent tumor cells were added to the endothelial monolayer in the presence of chemokine CXCL12 (100 ng/ml) in the lower compartment. After 24 hrs, cells remaining in the upper chamber were removed with a cotton swab and tumor cells having migrated through the endothelial monolayer to the lower face of the filter were lysed with water and quantified in a fluorescent microplate reader at wavelength 485/530 nm. Experiments were performed in triplicate.

### Cell migration

For Boyden chamber assays of cell migration, MDA-MB-231 cells (200.000/well) were pre-treated for 24 hrs with AngII (100 nM) or vehicle and were then seeded on the upper chamber of 8 µm-Transwell filters (Corning, NY, USA) either coated or not with 10 µg/ml matrigel (BD Biosciences), and allowed to migrate for 18 hrs in the presence of 10% FCS in the lower compartment. Cells migrating to the lower face of the filters were fixed in methanol, stained with crystal violet and counted under an inverted microscope. Experiments were performed in triplicate.

For wound healing assays, D3H2LN cells were pre-treated for 24 hrs with AngII (100 nM) or vehicle and were then grown to confluence in 24-well plates before cross-shape wounds were performed in the monolayer using a sterile 10 µl pipette tip. Wounds were registered by phase contrast microscopy immediately after scratching (T0) and after 16 hrs in serum-free medium (T16), and quantified using ImageJ software (http://rsb.info.nhi.gov/ij/). For each condition the ratio of wound closure at T16 relative to T0 was calculated.

### Gene array studies

Total RNA from MDA-MB-231 cells treated for 24 hrs with AngII (100 nM) or vehicle, was extracted using Trizol (Invitrogen) and analyzed with the Affymetrix Human Genome U133 Plus 2.0 Gene Chips (a genome wide array with 54674 probe sets targeting 19418 transcripts) as described [38]. Gene expression levels were normalized using the GC-RMA algorithm and flags were computed using MAS5. Quality assessment of the chips was performed with affyQCReport R package (R project for Statistical Computing [http://www.r-project.org/]). Each data set was derived from triplicates of biologically independent samples and compared using Student's *t* test. To estimate the false discovery rate the resulting p values were filtered at 5%. Microarray experiments were performed according to the MIAME consortium guidelines. Data have been submitted to MIAMEarray express under accession number E-MEXP-3470 and the release date is 2012-12-05. Data were submitted to Ingenuity Pathway Analysis (IPA) to model relationships among genes and proteins and to construct putative pathways and relevant biological processes (http://www.ingenuity.com).

### RT-PCR analysis

Total RNA was extracted from MDA-MB-231 cells treated as indicated, and cDNA was reverse-transcribed using oligo-dT and superscript RT (Invitrogen) as recommended by the manufacturer. PCR amplification (35 cycles, annealing temperature 55°C) was performed on 25 ng cDNA using oligonucleotide primers as follows: **MMP9-F** 5′AAG TAC TGG CGA TTC TCT GAG GG; **MMP9-R** 5′GGC TTT CTC TCG GTA CTG GAA GAC; **MMP2-F** 5′TTT TCT CGA ATC CAT GAT GG; MMP2-R 5′CTG GTG CAG CTC TCA TAT TT; **MMP3-F** 5′CCT GCT TTG TCC TTT GAT GC; **MMP3-R** 5′TGA GTC AAT CCC TGG AAA GTC; **GAPDH-F** 5′GGA GAA GGC TGG GGC; **GAPDH-R** 5′GAT GGC ATG GAC TGT GG.

### FACS analysis

MDA-MB-231 cells were treated for 24 hrs with AngII (100 nM) or vehicle and harvested in 1mM EDTA. Expression levels of ICAM-1 at the cell membrane were evaluated by FACS analysis using Cytomics TM FC500 (Beckman Coulter) after labeling with anti-ICAM-1 antibodies (R&D system).

### Gelatin zymography

For analysis of metalloprotease enzymatic activity, conditioned medium of MDA-MB-231 cells treated for 24 hrs with increasing concentrations of AngII, or lipopolysaccharide (LPS, 100 ng/ml) as positive control, were collected and loaded on gelatin (1 mg/ml)-containing SDS-PAGE run at 4°C (zymography gels) as described [23]. MMP9 activity was visualised as a clear band at 90 kDa after coomassie blue coloration, and quantified using ImageJ software.

### Statistical analysis

Statistical analyses were conducted using JMP-7 software. Data in bar graphs (mean +/− SEM) were analyzed using Student's t-test. p<0.05 was considered statistically significant.

## Supporting Information

Figure S1 (**A**). Quantification of the number of metastases per mouse at day 9. Shown are pooled results from 2 independent experiments, black squares and black triangles representing control (n = 15) and AngII-treated (n = 14) mice, respectively. (**B**). Quantification of the number of photons/s per mouse at day 9. Results are expressed as in (A). * p<0.05, ***p<0.001.
(TIF)

Figure S2 **MTT assay of D3H2LN cells proliferation following 24 hrs- pre-treatment with AngII** **(100 nM) or vehicle.** Shown is one representative experiment out of 3 performed in quadruplicate.
(TIF)

Table S1 **Shown are the 63 genes up-regulated by AngII (100 nM, 24 hrs) by 1.4-fold or more (p<0.05).** The genes are listed in alphabetical order, together with their main characteristics and known functions (description/ Gene pathway/ function column), differential regulation by AngII (fold column) and p value. (a): Genes connected to Angiotensinogen pathway AGT (as illustrated in Figure 3A) are indicated by an asterisk *.
(DOC)

Table S2 **Shown are the 39 genes down-regulated by AngII (100 nM, 24 hrs) by 1.4-fold or more (p<0.05).** The genes are listed in alphabetical order as indicated in Table S1. (a): Genes connected to Angiotensinogen pathway AGT (as illustrated in Figure 3A) are indicated by an asterisk *.
(DOC)

Table S3 **Genes regulated by AngII are classified according to their major functions namely Inflammation, Cell Proliferation and Apoptosis, Adhesion and Migration, Metabolism.** Genes with others functions appear in the “others” section. Number of genes is indicated under parenthesis. Up-regulated genes are indicated in bold whereas down-regulated genes are indicated in standard font.
(DOC)

Table S4 **Genes regulated by AngII are organized in four major pathways related to protein kinase signaling, small GTPases, Ubiquitin/proteasome and intracellular traffic.** Number of genes is indicated under parenthesis. Up-regulated genes are indicated in bold whereas down-regulated genes are indicated in standard font.
(DOC)
