# Supplementary figures and images for: Angiotensin II Facilitates Breast Cancer Cell Migration and Metastasis
Source: PLoS One. 2012 Apr 20;7(4):e35667. doi: 10.1371/journal.pone.0035667 (PMC3334979; doi:10.1371/journal.pone.0035667)

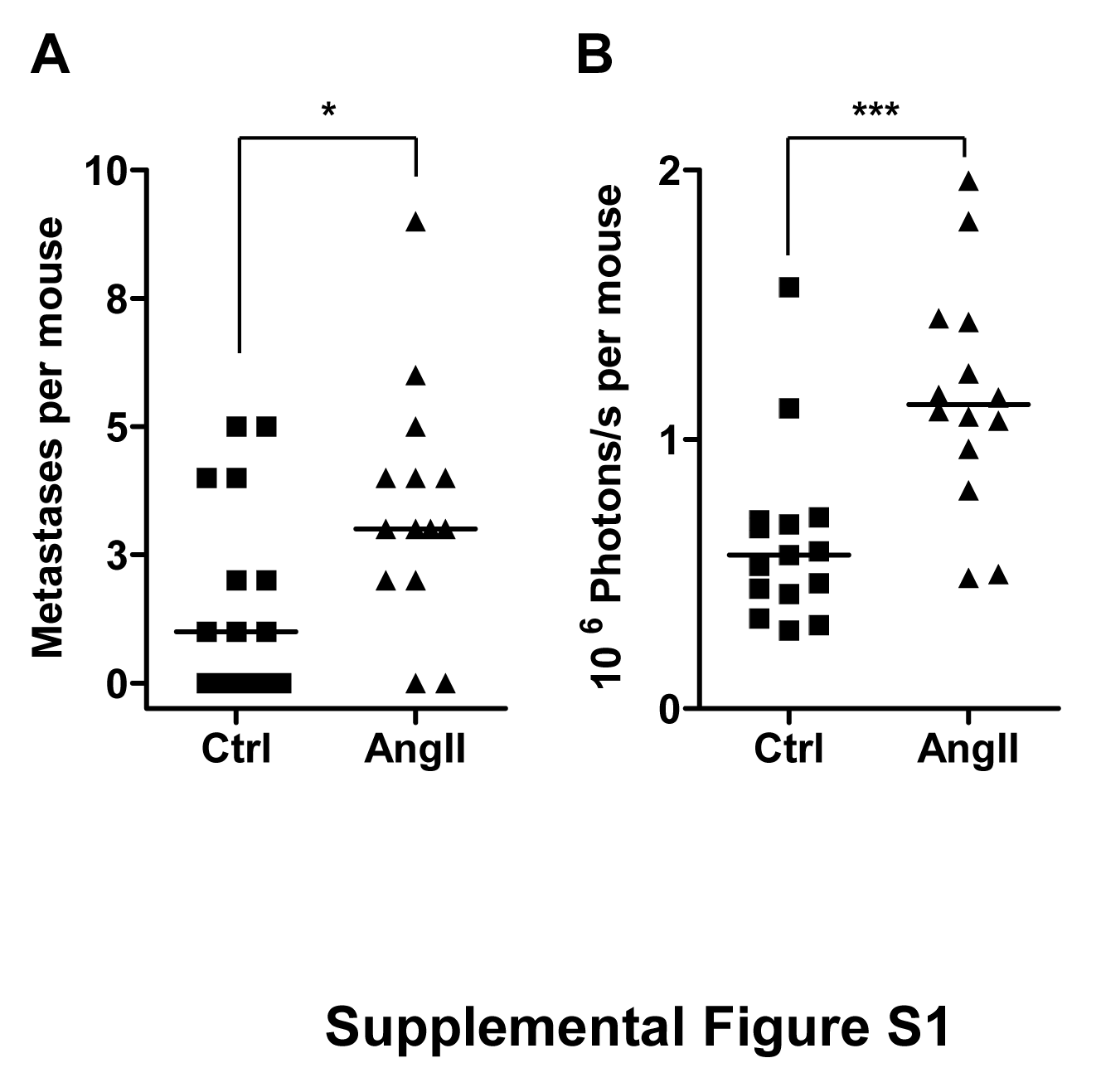

Supplement: Figure S1 — (A). Quantification of the number of metastases per mouse at day 9. Shown are pooled results from 2 independent experiments, black squares and black triangles representing control (n = 15) and AngII-treated (n = 14) mice, respectively. (B). Quantification of the number of photons/s per mouse at day 9. Results are expressed as in (A). * p<0.05, ***p<0.001. (TIF) [file pone.0035667.s001.tif]

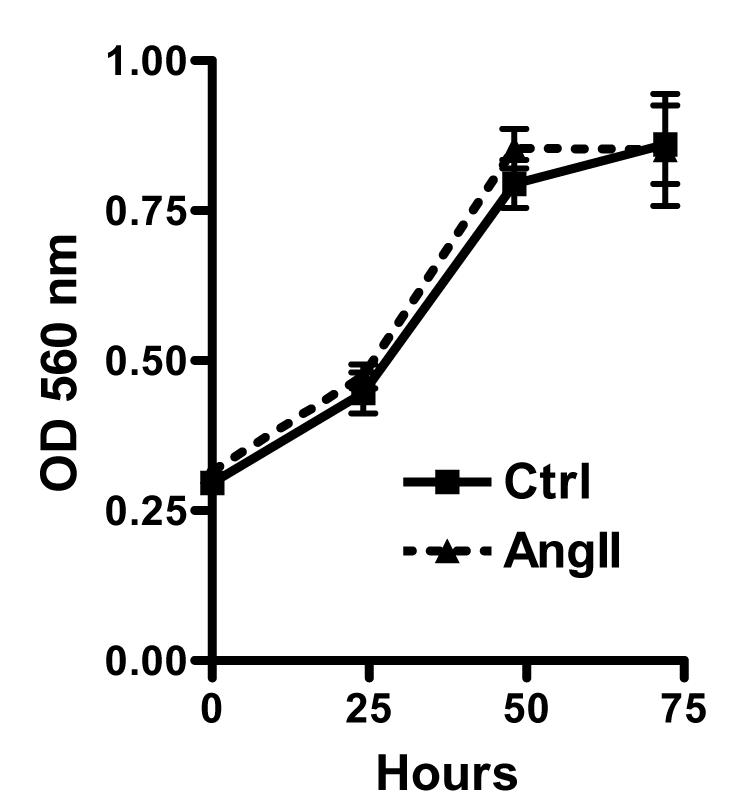

Supplement: Figure S2 — MTT assay of D3H2LN cells proliferation following 24 hrs- pre-treatment with AngII (100 nM) or vehicle. Shown is one representative experiment out of 3 performed in quadruplicate. (TIF) [file pone.0035667.s002.tif]
