# Supplementary material for: Angiotensin II Facilitates Breast Cancer Cell Migration and Metastasis
Source: PLoS One. 2012 Apr 20;7(4):e35667. doi: 10.1371/journal.pone.0035667 (PMC3334979; doi:10.1371/journal.pone.0035667)
Supplement: Table S1 — Shown are the 63 genes up-regulated by AngII (100 nM, 24 hrs) by 1.4-fold or more (p<0.05). The genes are listed in alphabetical order, together with their main characteristics and known functions (description/ Gene pathway/ function column), differential regulation by AngII (fold column) and p value. (a): Genes connected to Angiotensinogen pathway AGT (as illustrated in Figure 3A) are indicated by an asterisk *. (DOC) [file pone.0035667.s003.doc]

**Supplemental Table** S1. List of genes up-regulated by AngII in MDA-MB-231 cells.

| **Gene Symbol(a)** | **Gene Name** | **Description / Gene pathway / Function** | **fold** | **p value** |
| --- | --- | --- | --- | --- |
| ABCC10 | ATP-binding cassette, sub-family C (CFTR/MRP), member 10 | Transporter, Drug resistance, Metabolism | 1.55 | 0.035 |
| AKT1S1 | AKT1 substrate 1 (proline-rich) | Protein kinase signaling, Apoptosis | 2.15 | 0.025 |
| ALDH3B1 | Aldehyde dehydrogenase 3 family, member B1 | Enzyme, Metabolism, Inflammation | 1.57 | 0.021 |
| ALS2CL | ALS2 C-terminal like | Exchange factor, Small GTPase signaling, Proliferation, Intracellular traffic | 2.02 | 0.044 |
| ARFGAP1* | ADP-ribosylation factor GTPase activating protein 1 | Exchange factor, Small GTPase signaling, Intracellular traffic, Cell Adhesion/Migration | 1.60 | 0.022 |
| ARHGEF12* | Rho guanine nucleotide exchange factor (GEF) 12 | Exchange factor, Small GTPase signaling, Metabolism, Cell Adhesion/Migration | 3.03 | 0.034 |
| ARPC4 | Actin related protein 2/3 complex, subunit 4 | Actin binding protein, Small GTPase signaling, Intracellular traffic Cell Adhesion/Migration | 1.51 | 0.027 |
| ARRDC1 | Arrestin domain containing 1 | Arrestin-related trafficking, Cell Adhesion/Migration | 1.82 | 0.041 |
| ATAD3A | ATPase family, AAA domain containing 3A | ATP binding protein, Apoptosis | 4.55 | 0.032 |
| ATXN7L3 | Ataxin 7-like 3 | Zn finger, Trancriptional regulator, Histone ubiquitination | 1.48 | 0.045 |
| BSG* | Basigin (EMMPRIN) | Receptor, Protein kinase signaling, Cell Adhesion/Migration, Inflammation, Metabolism | 1.81 | 0.042 |
| CARD10 | Caspase recruitment domain family, member 10 | Guanylate kinase family member, Protein kinase signaling, Cell Growth and Apoptosis, Cell Adhesion/Migration | 1.55 | 0.035 |
| CDKN1C* | Cyclin-dependent kinase inhibitor 1C (p57, Kip2) | Kinase, Proliferation, Actin dynamics, Neuronal differentiation, Tumor invasion and metastasis | 1.50 | 0.017 |
| CYB5R2 | Cytochrome b5 reductase 2 | Enzyme, NADH redox activity, Metabolism | 1.81 | 0.015 |
| DDA1 | DET1 and DDB1 associated 1 | Autophagy, Ubiquitination, Tumor invasion and Metastasis | 1.61 | 0.048 |
| DLGAP4 | Discs, large (Drosophila) homolog-associated protein 4 | Guanylate kinase, Receptor interacting protein | 1.44 | 0.040 |
| DOK1* | Docking protein (downstream of tyrosine kinase 1) | Scaffold protein, Receptor tyrosine kinase signaling, Proliferation, Cell Adhesion/Migration | 2.06 | 0.004 |
| DOLK | Dolichol kinase | Kinase, Glycosylation, Metabolism | 1.55 | 0.040 |
| EFNB3 | Ephrin-B3 | Receptor tyrosine kinase, Small GTPase and Protein kinase signaling, Cell Growth and Apoptosis, Cell Adhesion/Migration | 1.43 | 0.047 |
| EIF5A | Eukaryotic translation initiation factor 5A | Translation factor, Cell Growth and Apoptosis, Metabolism, Cell Adhesion/Migration | 1.73 | 0.048 |
| FBXL19 | F-box and leucine-rich repeat protein 19 | Enzyme, F-Box protein family, Ubiquitin/Proteasome pathway | 2.04 | 0.037 |
| FMNL3 | Formin-like 3 | Actin binding protein, Small GTPase signaling, Cell Adhesion/Migration | 1.48 | 0.049 |
| FRMD4A | FERM domain containing 4A | Actin interacting domain, Small GTPase signaling, Actin dynamics, Epithelial polarity, Cell Adhesion/Migration | 1.43 | 0.027 |
| FUT4 | Fucosyltransferase 4 (alpha (1,3), myeloid-specific) | Enzyme, Protein kinase signaling, Selectin-related interactions, Cell Adhesion/Migration, Inflammation, Metabolism | 1.82 | 0.043 |
| GNG7 | Guanine nucleotide binding protein (G protein), gamma 7 | Protein G gamma subunit, GPCR signalling, Cell Growth | 1.62 | 0.011 |
| HMG20B | High-mobility group 20B | Transcription regulator, DNA binding, BRCA2 interaction | 1.87 | 0.018 |
| ICAM1* | Intercellular adhesion molecule 1 | Transmembrane cell adhesion molecule, Inflammation, Cell Adhesion/Migration | 1.43 | 0.050 |
| IGF1R* | Insulin-like growth factor 1 receptor | Receptor, Protein kinase signaling, Cell Growth and Apoptosis, Inflammation | 1.71 | 0.050 |
| IL17RA | Interleukin 17 receptor A | Receptor, Protein kinase signaling, Inflammation | 1.60 | 0.047 |
| ITGB2* | Integrin, beta 2 (complement component 3 receptor 3 and 4 subunit) | Receptor, Small GTPase signaling, Inflammation, Cell Adhesion/Migration | 1.50 | 0.032 |
| KDELR1* | KDEL (Lys-Asp-Glu-Leu) endoplasmic reticulum protein retention receptor 1 | Receptor/Transporter, Protein kinase signaling, Small GTPase signaling, Intracellular traffic, ER stress response | 1.85 | 0.030 |
| MAN1B1 | Mannosidase, alpha, class 1B, member 1 | Enzyme, Metabolism, Intracellular traffic, Proteasome pathway | 1.89 | 0.046 |
| MAP2K7* | Mitogen-activated protein kinase kinase 7 | Kinase, Protein kinase signaling, Cell Growth and Apoptosis, Inflammation | 1.46 | 0.048 |
| MAP4K2 | Mitogen-activated protein kinase kinase kinase kinase 2 | Kinase, Protein kinase signaling, Inflammation | 2.20 | 0.046 |
| MKNK2* | MAP kinase interacting serine/threonine kinase 2 | Calcium/calmodulin dependent Protein kinase signaling,Translation, Metabolism, Cell Proliferation, Inflammation, Proteasome pathway | 1.89 | 0.022 |
| MRPS18A | Mitochondrial ribosomal protein S18A | Mitochondrial, ribosomal, Protein synthesis | 1.77 | 0.040 |
| MYH11 | Myosin, heavy chain 11, smooth muscle | Actin binding protein, TGFbeta pathway, Motor activity, Cell Proliferation, Cell Adhesion/Migration | 1.66 | 0.024 |
| MYO1C* | Myosin IC | Actin based molecular motor, Cell Proliferation, Intracellular traffic, Cell Adhesion/Migration | 1.49 | 0.036 |
| NAT8L | N-acetyltransferase 8-like (GCN5-related, putative) | Enzyme, Nacetyl methyltransferase superfamily, Membrane-bound protein, neuron-specific, Metabolism | 1.74 | 0.045 |
| NEK8 | NIMA (never in mitosis gene a)- related kinase 8 | Kinase, Cell Cycle progression, Cell proliferation, Ciliogenesis | 1.60 | 0.042 |
| OTUD5 | OTU domain containing 5 | Cysteine protease, Deubiquitinase, Innate immune response, Inflammation, Proteasome pathway | 1.55 | 0.046 |
| PCGF1 | Polycomb group ring finger 1 | Transcriptional regulator, Development, Ubiquitin/Proteasome pathway | 1.51 | 0.036 |
| PCTK1 | PCTAIRE protein kinase 1 | Cyclin dependent kinase Cdk16, Protein kinase signaling, Intracellular traffic, Exocytosis | 1.56 | 0.029 |
| PRIC285 | Peroxisomal proliferator-activated receptor A interacting complex 285 | Zinc finger, Transcription regulator, DNA helicase, Metabolism, Inflammation | 1.56 | 0.004 |
| PYCR2 | Pyrroline-5-carboxylate reductase family, member 2 | Enzyme, Metabolism | 1.44 | 0.023 |
| RAB4B | RAB4B, member RAS oncogene family | Enzyme, Small GTPase signaling, Intracellular traffic, Glut4 transport, Metabolism, Motility | 1.56 | 0.036 |
| RASGRF1* | Ras protein-specific guanine nucleotide-releasing factor 1 | Exchange factor, Small GTPase signaling, Protein kinase signaling, Cell Proliferation, Cell Adhesion/Migration | 2.90 | 0.001 |
| SEMA6B | Sema domain, transmembrane domain (TM), and cytoplasmic domain, (semaphorin) 6B | Transmembrane receptor, Axon guidance, Cell Adhesion/Migration | 2.64 | 0.005 |
| SHB | Src homology 2 domain containing adaptor protein B | Adaptator protein, Protein kinase and Small GTPase signaling, Cell Growth and Apoptosis, Cell Adhesion/Migration, Inflammation | 1.50 | 0.026 |
| SIX2 | SIX homeobox 2 | Transcription factor, Cortisol Secretion, Metabolism | 1.48 | 0.043 |
| SLC2A4RG | SLC2A4 regulator | Transcription factor, Glut4 enhancer, Metabolism | 1.51 | 0.039 |
| SRGAP1 | SLIT-ROBO Rho GTPase activating protein 1 | Small GTPase signaling, Neuronal Migration | 2.03 | 0.047 |
| STX10 | Syntaxin 10 | SNAP receptor activity, Vesicular transport, Intracellular traffic | 1.47 | 0.023 |
| TBC1D10A | TBC1 domain family, member 10A | Exchange factor, Small GTPase signaling, Intracellular traffic | 1.83 | 0.020 |
| THRA | Thyroid hormone receptor, alpha | Nuclear hormone receptor, Metabolism | 1.51 | 0.022 |
| TNFRSF12A* | Tumor necrosis factor receptor superfamily, member 12A | Membrane receptor, Protein kinase signaling, Apoptosis, Inflammation | 1.86 | 0.045 |
| TRAF3IP2 | TRAF3 interacting protein 2 | TNF receptor pathway, Protein kinase signaling, Apoptosis, Inflammation, Proteasome pathway | 1.85 | 0.034 |
| TSPAN4 | Tetraspanin 4 | Transmembrane protein, Cell Growth and Apoptosis, Cell Adhesion/Migration | 1.98 | 0.034 |
| UBE2M | Ubiquitin-conjugating enzyme E2M (UBC12 homolog) | Enzyme, Ubiquitin/Proteasome pathway, Cell Proliferation and Apoptosis | 1.78 | 0.032 |
| UBE2R2 | Ubiquitin-conjugating enzyme E2R 2 | Enzyme, Ubiquitin/Proteasome pathway, Cell Growth | 1.50 | 0.024 |
| UBXN11 | UBX domain protein 11 | Small GTPase signaling, Cell Adhesion/Migration Ubiquitin/Proteasome pathway | 1.48 | 0.015 |
| VPS37D | Vacuolar protein sorting 37 homolog D | Intracellular traffic | 2.05 | 0.047 |
| WIZ | Widely interspaced zinc finger motifs | Zinc finger, Nuclear co-repressor, Histone methylation | 2.06 | 0.025 |
