# Supplementary material for: Angiotensin II Facilitates Breast Cancer Cell Migration and Metastasis
Source: PLoS One. 2012 Apr 20;7(4):e35667. doi: 10.1371/journal.pone.0035667 (PMC3334979; doi:10.1371/journal.pone.0035667)
Supplement: Table S2 — Shown are the 39 genes down-regulated by AngII (100 nM, 24 hrs) by 1.4-fold or more (p<0.05). The genes are listed in alphabetical order as indicated in Table S1. (a): Genes connected to Angiotensinogen pathway AGT (as illustrated in Figure 3A) are indicated by an asterisk *. (DOC) [file pone.0035667.s004.doc]

**Supplemental** Table S2. List of genes down-regulated by AngII in MDA-MB-231 cells.

| **Gene**  **Symbol (a)** | **Gene Name** | **Description / Gene pathway / Function** | **fold** | **p value** |
| --- | --- | --- | --- | --- |
| ANAPC10* | Anaphase promoting complex subunit 10 | Enzyme, Cell Proliferation and Apoptosis, Ubiquitination | -2.21 | 0.041 |
| ARL17 | ADP-ribosylation factor-like 17 | Metabolism | -1.72 | 0.040 |
| B4GALT4 | UDP-Gal:betaGlcNAc beta 1,4- galactosyltransferase, polypeptide 4 | Enzyme, Membrane-bound protein, Metabolism | -1.42 | 0.038 |
| BTBD3 | BTB (POZ) domain containing 3 | Interacts with PlexinB3 | -1.42 | 0.035 |
| COG5 | Component of oligomeric golgi complex 5 | Transporter, Intracellular traffic | -2.14 | 0.026 |
| DOCK5 | Dedicator of cytokinesis 5 | Exchange factor, Small GTPase signaling, Cell Adhesion/Migration | -1.57 | 0.043 |
| DYRK2 | Dual-specificity tyrosine-(Y)-phosphorylation regulated kinase 2 | Kinase, Protein kinase signaling, Cell Proliferation, | -1.69 | 0.048 |
| EIF2S3 | Eukaryotic translation initiation factor 2, subunit 3 gamma, 52kDa | Translation regulator, Protein synthesis, Cell Proliferation | -2.08 | 0.024 |
| EXOC8 | Exocyst complex component 8 | Cell Adhesion/Migration, Intracellular traffic | -1.52 | 0.032 |
| FBXO45 | F-box protein 45 | Ubiquitination | -1.69 | 0.005 |
| FGFR1OP2 | FGFR1 oncogene partner 2 | Cell Proliferation, Cell Differentiation | -1.75 | 0.045 |
| HCFC2 | Host cell factor C2 | Transcription regulator, Cell Proliferation | -1.86 | 0.049 |
| IDH3A | Isocitrate dehydrogenase 3 (NAD+) alpha | Enzyme, Metabolism | -2.02 | 0.003 |
| IRAK3 | Interleukin-1 receptor-associated kinase 3 | Transmembrane receptor, Protein kinase signaling, Inflammation | -1.81 | 0.037 |
| KIF1B | Kinesin family member 1B | Transporter, Intracellular traffic, Cell Adhesion/Migration | -2.16 | 0.040 |
| KPNA1 | Karyopherin alpha 1 (importin alpha 5) | Transporter, Intracellular traffic, Inflammation | -1.47 | 0.026 |
| MAP7D3 | MAP7 domain containing 3 | Microtubule associated protein 7, Cell Adhesion/Migration | -2.51 | 0.029 |
| MAPK1* | Mitogen-activated protein kinase 1 | Kinase, Protein kinase signaling, Transcription regulation, Cell Proliferation | -1.50 | 0.038 |
| MITF | Microphthalmia-associated transcription factor | Transcription factor, Cell differentiation, Cell Proliferation and Apoptosis | -1.47 | 0.039 |
| MSRB2 | Methionine sulfoxide reductase B2 | Transcription regulator, Metabolism | -1.76 | 0.021 |
| NDUFS1* | NADH dehydrogenase (ubiquinone) Fe-S protein 1, 75kDa (NADH-coenzyme Q reductase) | Enzyme, Metabolism | -1.39 | 0.013 |
| OSGEPL1 | O-sialoglycoprotein endopeptidase-like 1 | Enzyme, Metabolism | -1.50 | 0.032 |
| PAG1 | phosphoprotein associated with glycosphingolipid microdomains 1 | Transmembrane protein, Protein kinase signaling, Inflammation | -1.66 | 0.038 |
| PAWR* | PRKC, apoptosis, WT1, regulator | Transcription regulator, Cell Proliferation and Apoptosis | -1.99 | 0.011 |
| PTPN21 | Protein tyrosine phosphatase, non-receptor type 21 | Protein tyrosine phosphatase, Cell Proliferation and Apoptosis, Cell Differentiation | -1.52 | 0.004 |
| RALB | V-ral simian leukemia viral oncogene homolog B (ras related; GTP binding protein) | Enzyme, Small GTPase signaling, Cell Proliferation and Apoptosis, Cell Adhesion/Migration | -1.50 | 0.026 |
| RGS2* | Regulator of G-protein signaling 2, 24kDa | GTPase activating protein, Small GTPase signaling, Cell Proliferation and Apoptosis | -1.49 | 0.023 |
| RNF144B | Ring finger protein 144B | Enzyme, Ubiquitination, Metabolism | -1.46 | 0.009 |
| RTTN | Rotatin | Development | -1.90 | 0.015 |
| SFRS3 | Splicing factor, arginine/serine-rich 3 | Splicing factor, Gene expression | -1.81 | 0.032 |
| SGMS2 | Sphingomyelin synthase 2 | Enzyme, Metabolism, Cell Growth and Apoptosis | -1.50 | 0.045 |
| SLC40A1 | Solute carrier family 40 (iron-regulated transporter), member 1 | Transporter, Metabolism | -1.91 | 0.023 |
| SMAD2* | SMAD family member 2 | Transcription regulator, Cell Proliferation and Apoptosis | -2.48 | 0.006 |
| SYNE1 | Spectrin repeat containing, nuclear envelope 1 | Nuclear membrane protein, Cytoskeletal anchoring, Differentiation, Cell Adhesion/Migration | -2.70 | 0.013 |
| UBE2H | Ubiquitin-conjugating enzyme E2H (UBC8 homolog, yeast) | Enzyme, Ubiquitination | -1.77 | 0.025 |
| ZFP82 | Zinc finger protein 82 homolog (mouse) | Zinc finger protein,Transcription regulator | -1.93 | 0.001 |
| ZNF354B | Zinc finger protein 354B | Zinc finger protein, Transcription regulator | -2.36 | 0.041 |
| ZNF57 | Zinc finger protein 57 | Zinc finger protein, Transcription regulator | -2.24 | 0.022 |
| ZRANB1 | Zinc finger, RAN-binding domain containing 1 | Peptidase, Metabolism, Inflammation | -1.40 | 0.034 |
