# Supplementary material for: Angiotensin II Facilitates Breast Cancer Cell Migration and Metastasis
Source: PLoS One. 2012 Apr 20;7(4):e35667. doi: 10.1371/journal.pone.0035667 (PMC3334979; doi:10.1371/journal.pone.0035667)
Supplement: Table S3 — Genes regulated by AngII are classified according to their major functions namely Inflammation, Cell Proliferation and Apoptosis, Adhesion and Migration, Metabolism. Genes with others functions appear in the “others” section. Number of genes is indicated under parenthesis. Up-regulated genes are indicated in bold whereas down-regulated genes are indicated in standard font. (DOC) [file pone.0035667.s005.doc]

**Supplemental Table S3: Genes regulated by AngII classified according to their functions**

| Inflammation  (18) | **ALDH3B1, BSG, FUT4, ICAM1, IGF1R**, **IL17RA,** IRAK3, **ITGB2**, KPNA1, **MAP2K7**, **MAP4K2**, **OTUD5,** PAG1, **PRIC285**, **SHB,** **TNFRSF12A**, **TRAF3IP2**, ZRANB1 |
| --- | --- |
| Cell Proliferation and Apoptosis (32) | **AKT1S1**, **ALS2CL,** ANAPC10, **ATAD3A**, **CDKN1C, DOK1**, DYRK2, **EFNB3**, EIF2S3, FGFR10P2, **GNG7,** HCFC2, **IGF1R**, MAPK1, **MAP2K7,** MITF, **MYH11, MYOC1,** **NEK8,** PAWR, PTPN21, RALB, **RASGRF1,** RGS2, SGMS2, **SHB,** SMAD2, **TNFRSF12A**, **TRAF3IP2**, **TSPAN4**, **UBE2M, UBE2R2** |
| Adhesion and Migration  (27) | **ARFGAP1, ARHGEF12**, **ARPC4, ARRDC1, BSG**, **DOK1,** DOCK5, EXOC8, **EFNB3,** **FMNL3, FRMD4A, FUT4,** **ICAM1, ITGB2,** KIF1B, MAP7D3, **MYH11, MYOC1, RAB4B,** RALB, **RASGRF1**, **SEMA6B, SHB**, **SRGAP1**, SYNE1, **TSPAN4**, **UBXN11** |
| Metabolism  (25) | **ABCC10, ALDH3B1, ARHGEF12,** ARL17, B4GALT4, **BSG, CYBR5, DOLK, EIF5A, FUT4,** IDH3A, MSRB2, **NATL8,** NDUFS1, OSGEPL1, **PRIC285**, **PYCR2**, **RAB4B**, RNF144B, SGMS2, **SIX2**, **SLC2A4RG,** SLC40A1, **THRA**, ZRANB1 |
| Others  (23) | **ATXN7L3**, BTBD3, COG5, **DDA1**, **DLGAP4,** **FBXL19**, FBX045, **HMG20B,** **KDELR1**, **MAN1B1, MRPS18A, PCGF1, PCTK1**, RTTN, SFRS3, **STX10, TBC1D10A**, **UBE2H, VPS37D**, **WIZ,** ZFP82, ZNFF354B, ZNF57 |
