# Supplementary material for: Angiotensin II Facilitates Breast Cancer Cell Migration and Metastasis
Source: PLoS One. 2012 Apr 20;7(4):e35667. doi: 10.1371/journal.pone.0035667 (PMC3334979; doi:10.1371/journal.pone.0035667)
Supplement: Table S4 — Genes regulated by AngII are organized in four major pathways related to protein kinase signaling, small GTPases, Ubiquitin/proteasome and intracellular traffic. Number of genes is indicated under parenthesis. Up-regulated genes are indicated in bold whereas down-regulated genes are indicated in standard font. (DOC) [file pone.0035667.s006.doc]

| Protein Kinase Signaling  (21) | **AKTS1, BSG, CARD10, DOK1,** DYRK2, **EFNB3, FUT4, IGF1R, IL17RA,** IRAK3, **KDELR1,** MAPK1, **MAP2K7, MAP4K2, MKNK2,** PAG1, **PCTK1, RASGRF1, SHB, TNFRSF12A, TRAF3IP2** |
| --- | --- |
| Small GTPase Signaling  (18) | **ALS2CL, ARFGAP1, ARHGEF12, ARPC4,** DOCK5, **EFNB3, FMNL3, FRMD4A, ITGB2, KDELR1, RAB4B,** RALB, **RASGRF1,** RGS2, **SHB, SRGAP1, TBC1D10A, UBXN11** |
| Ubiquitin/Proteasome  (13) | ANAPC10, **DDA1, FBXL19,** FBXO45, **MAN1B1, MKNK2, OTUD5, PCGF1,** RNF144B, UBE2H, **UBE2M, UBE2R2, UBXN11** |
| Intracellular Traffic  (10) | COG5, EXOC8, **KDELR1,** KIF1B, KPNA1, **MAN1B1, PCTK1, RAB4B, STX10, VPS37D** |

**Supplemental Table S4: Genes regulated by AngII classified according to their signaling pathways**
